# Prebiotic Structural Diversity Shapes Gut Microbial Diversity, Community Composition, and Metabolic Activity In Vitro

**DOI:** 10.3390/foods14213774

**Published:** 2025-11-04

**Authors:** Yousi Fu, Yali Wang, Junnan Zhang, Jianlin Ren, Baishan Fang

**Affiliations:** 1 Department of Chemical and Biochemical Engineering, College of Chemistry and Chemical Engineering, Xiamen University, Xiamen 361005, China; fuyousi@msu.edu (Y.F.); wangyal1@msu.edu (Y.W.); hello_zjn@163.com (J.Z.); 2 Department of Gastroenterology, The National Key Clinical Specialty, Zhongshan Hospital of Xiamen University, School of Medicine, Xiamen University, Xiamen 361004, China

**Keywords:** gut microbiota, Prebiotics, short chain fatty acids, in vitro culture

## Abstract

Prebiotics are selectively utilized substrates that modulate gut microbiota and host health, yet different prebiotic structures may elicit distinct ecological and metabolic responses. In this study, we investigated the effects of five structurally diverse prebiotics—isomaltooligosaccharides (IMO), arabinogalactans (AG), pectin, inulin, and stachyose—on human gut microbiota via a 24 h in vitro anaerobic culture with healthy donors’ gut microbiota. Microbial community dynamics were profiled by 16S rRNA gene sequencing, and short-chain fatty acids (SCFAs) production was analyzed. All treatments resulted in decreased α-diversity compared with baseline, with pectin most effectively preserving microbial richness and evenness, whereas stachyose led to the greatest reduction. Community composition and functional profiles shifted in a substrate-specific manner, with AG promoting *Bacteroidaceae*, IMO stimulating *Lachnospiraceae* and *Faecalibacterium*, and pectin supporting balanced microbial structures and SCFA production. Pectin, IMO, and inulin enhanced butyrate levels, whereas AG and pectin promoted propionate formation. These findings demonstrate that prebiotic structural differences strongly shape gut microbial ecology and metabolism, providing a mechanistic basis for rationally selecting and combining prebiotics to beneficially modulate the gut microbiota.

## 1. Introduction

The human gut harbors a highly complex and dynamic microbial ecosystem, containing billions of microorganisms including bacteria, viruses, fungi, and archaea [1]. These gut microbes interact extensively with the host and play crucial roles in digestion, nutrient metabolism, immune function, and even disease development [2]. Accumulating evidence indicates that gut microbiota dysbiosis—an imbalance in the composition or function of gut microbial communities—is closely linked to various diseases, including inflammatory bowel disease (IBD), metabolic syndrome, obesity, liver disorders, and neuropsychiatric conditions [3,4,5,6]. In patients with IBD, gut microbiota dysbiosis is typically characterized by a marked reduction in the families *Lachnospiraceae* and *Ruminococcaceae*, together with an overrepresentation of the *Enterobacteriaceae* family [7]. In obese individuals, the gut microbiota is characterized by reduced microbial diversity and depletion of beneficial bacteria such as *Akkermansia muciniphila*, and *Bifidobacterium longum* [8]. Therefore, modulating gut microbiota structure and function has garnered increasing attention for promoting human health and preventing disease.

Among the numerous microbiota-derived metabolites, short-chain fatty acids (SCFAs), primarily acetic acid (acetate), propionic acid (propionate) and butyric acid (butyrate), have marked effects on host physiological functions [9]. SCFAs are generated through bacterial fermentation of indigestible carbohydrates and contribute significantly to host energy metabolism and gut homeostasis [10]. Acetate can be produced by *Bifidobacterium* spp., propionate can be generated by *Akkermansia muciniphila*, and butyrate can be produced by members of the *Lachnospiraceae* and *Ruminococcaceae* families, including *Faecalibacterium prausnitzii* [11,12]. In the human colon, acetate accounts for 60%, while propionate and butyrate each account for 20% [13]. Among SCFAs, butyrate is the most extensively studied immune system modulator [12]. As the preferred energy substrate for colonocytes, butyrate plays a crucial role in maintaining gut epithelial barrier function, regulating immune responses, and exerting anti-inflammatory effects [12,14,15]. Reduced levels of butyrate are frequently observed in patients with IBD, highlighting the significance of microbial fermentation activity in health and disease [16].

Prebiotics are defined as “substrates that are selectively utilized by host microorganisms conferring a health benefit” by International Scientific Association of Probiotics and Prebiotics (ISAPP) [17]. They are typically non-digestible carbohydrates, such as oligosaccharides and soluble fibers, that escape digestion in the upper gastrogut tract and reach the colon intact, where they are metabolized by specific gut microbiome [18]. In several clinical studies, supplementing with prebiotics has been shown to promote the growth and activity of beneficial microbiome, regulate microbial metabolic outputs, and affect host physiological functions [19,20]. However, prebiotics exhibit chemically diverse, differing in molecular size, glycosidic linkage type, and branching patterns. These characteristics determine their fermentation sites, degradation rates, and selectivity toward gut microbiome [21]. Consequently, different prebiotics may exert distinct effects on gut microbiota composition and metabolic profiles. For instance, inulin and fructooligosaccharides have been reported to selectively stimulate *Bifidobacterium* and *Lactobacillus* spp [17], while pectin favors the growth of *Bacteroides* [22].

Despite these advances, comparative studies systematically evaluating how different prebiotics shape human gut microbiota under controlled conditions remain limited. In particular, most in vivo studies are influenced by inter-individual variability, dietary background, and host factors, making it difficult to disentangle the direct effects of prebiotics on the microbial community itself [23,24]. In vitro culture systems offer a controlled platform to investigate the ecological and metabolic responses of gut microbiota to specific substrates, allowing for time-resolved analysis of community dynamics and metabolic outputs [25,26].

In this study, we employed an anaerobic batch-culture model inoculated with gut microbiota from healthy donors to investigate the effects of five prebiotics—isomaltooligosaccharides (IMO), arabinogalactans (AG), pectin, inulin, and stachyose—on gut microbial diversity, community structure, and SCFAs metabolism over a 24 h incubation period. Microbial community shifts were analyzed by 16S rRNA gene sequencing, and metabolic outputs were assessed by liquid chromatography. This comparative screening provides insights into how substrate diversity shapes gut microbial ecosystems and offers a rational basis for designing targeted prebiotic combinations and optimizing in vitro culture models.

## 2. Materials and Methods

### 2.1. Prebiotics and Chemicals

Five prebiotics were used for this study: isomaltooligosaccharides (IMO) (Baolingbao Biology, Dezhou, China), arabinogalactans (AG) (Sigma–Aldrich, St. Louis, MI, USA), pectin (Sangon Biotech, Shanghai, China), inulin (Beneo Orafti, Oreye, Belgium), and stachyose (Danisco, Suzhou, China). All prebiotics were food-grade or analytical-grade purity. Other chemicals included yeast extract, peptone, sodium chloride, potassium phosphate monobasic and dibasic, sodium bicarbonate, calcium chloride hexahydrate, magnesium sulfate heptahydrate, hemin, Tween 80, vitamin K_1_, bile salts, and L-cysteine hydrochloride (all from Sangon Biotech, Shanghai, China). Acetic acid, propionic acid, and butyric acid standards were obtained from Sigma-Aldrich for SCFAs quantification.

### 2.2. Gut Microbiota Preparation

Gut microbiota samples were obtained from three healthy donors at Zhongshan Hospital of Xiamen University. None of the donors had received antibiotics within three months prior to sampling or had a history of gastrogut disease. Samples were pooled after collection to minimize inter-individual variation. This study did not involve human study and human tissue. All donors provided written informed consent prior to participation. The procedures for sample handling, and informed consent followed our previously studies [27,28,29].

### 2.3. In Vitro Gut Microbiota Culture

In vitro anaerobic culture was conducted to investigate the effects of different prebiotics on human gut microbiota. For each treatment, 135 mL of basal culture medium was added to a 250 mL anaerobic bottle. The basal medium contained (per liter): 2 g of yeast extract, 2 g of peptone water, 0.1 g of NaCl, 0.04 g of KH_2_PO_4_, 0.04 g of K_2_HPO_4_, 2 g of NaHCO_3_, 0.01 g of CaCl_2_·6H_2_O, 0.01 g of MgSO_4_·7H_2_O, 50 mg of Hemin, 2 mL of Tween 80, 10 μL of Vitamin K1, 0.5 g of bile salts, 0.5 g of L-cysteine). The pH of the medium was adjusted to 7.0 ± 0.1, and nitrogen gas was bubbled for 15 min to maintain anaerobiosis. The medium was sterilized by autoclaving at 121 °C for 15 min. After cooling, each prebiotic was filter-sterilized and added to the basal medium at a final concentration of 2 g/L. 15 mL of gut microbiota was inoculated into each bottle. Cultures were incubated anaerobically at 37 °C with shaking at 150 rpm for 24 h. Samples were collected at 0, 4, 8, 12, and 24 h, and aliquoted for downstream DNA extraction and metabolite analysis. All cultures were performed in triplicate for each prebiotic. Samples were stored at −80 °C until further analysis.

### 2.4. DNA Extraction and 16S rRNA Gene Sequencing

Total microbial DNA was extracted from 200 μL of fermentation samples using the Lysing Matrix E bead-beating protocol (MP Biomedicals, Santa Ana, CA, USA) combined with the QIAGEN Stool DNA extraction kit, following the manufacturer’s instructions with slight modifications. The V1–V3 hypervariable regions of the bacterial 16S rRNA gene were amplified using primers F27 (5′-AGAGTTTGATCCTGGCTCAG-3′) and R534 (5′-ATTACCGCGGCTGCTGG-3′). PCR amplification was performed in 25 μL reactions containing 1 μL Phanta UC polymerase, 2 μL dNTPs (2.5 mM each), 5 μL 5× buffer, 1 μL of each primer (10 μM), 0.2 μL uracil-N-glycosylase, and approximately 20 ng of template DNA. Cycling conditions were: 95 °C for 3 min; 30 cycles of 95 °C for 20 s, 58 °C for 30 s, 72 °C for 2 min; and a final extension at 72 °C for 5 min. Amplicons were sequenced using the Illumina MiSeq platform (Illumina, San Diego, CA, USA).

### 2.5. Sequencing Data Analysis

Paired-end reads were merged using FLASH (version 1.2.11), and sequence processing was performed with QIIME (Quantitative Insights Into Microbial Ecology, version 1.8.0) [30]. Quality filtering and chimera removal were applied, and sequences were clustered into operational taxonomic units (OTUs) at 97% similarity. Taxonomic classification was performed against the Greengenes (version 13.8) reference database.

Alpha diversity indices (Shannon index, observed OTUs) were calculated in QIIME. Beta diversity was assessed based on Bray–Curtis distances, and ordination was visualized by constrained principal coordinate analysis (CPCoA) using in the vegan package (version 2.5-7). Permutational multivariate analysis of variance (PERMANOVA) was performed using the “adonis” function in the vegan package (version 2.5-7) to evaluate differences in community structure among groups and across time points. Statistical significance was assessed based on 999 permutations. Functional prediction of microbial communities was conducted using PICRUSt (Phylogenetic Investigation of Communities by Reconstruction of Unobserved States, version 1.1.4) to infer KEGG pathway profiles.

### 2.6. Short-Chain Fatty Acids Determination

For SCFAs measurement, 1 mL of culture broth was centrifuged at 10,000 × g for 10 min at 4 °C. The supernatant was filtered through a 0.22 μm membrane and analyzed by high-performance liquid chromatography (HPLC; Agilent 1200, Santa Clara, CA, USA) equipped with an Aminex HPX-87H column (Bio-Rad, Hercules, CA, USA) and a refractive index detector. The mobile phase was 5 mM H_2_SO_4_ at a flow rate of 0.5 mL/min, with a column temperature of 65 °C and detector temperature of 45 °C. The injection volume was 20 μL. Concentrations of acetate, propionate, and butyrate were quantified using external calibration curves prepared with authentic standards.

### 2.7. Statistical Analysis

All data were expressed as mean ± standard deviation (SD) of three independent cultures. Statistical analyses were conducted using R (version 3.5.0). Within each prebiotic treatment group, differences between 0 h baseline and 24 h were evaluated using paired *t*-tests or Wilcoxon signed-rank tests, depending on data distribution. One-way analysis of variance (ANOVA) followed by Tukey’s post hoc test was used for multiple comparisons where appropriate. A *p*-value < 0.05 was considered statistically significant.

## 3. Results

### 3.1. Impacts of Prebiotics on Microbial Diversity

To evaluate how different prebiotics modulate gut microbiota, we performed 24 h in vitro anaerobic cultures using pooled human gut microbiota as the inoculum. Five prebiotic substrates—isomaltooligosaccharides (IMO), arabinogalactans (AG), pectin, inulin, and stachyose—were tested in parallel. Microbial community dynamics were monitored at multiple time points, and α-diversity was assessed based on 16S rRNA gene sequencing.

**Figure 1:**
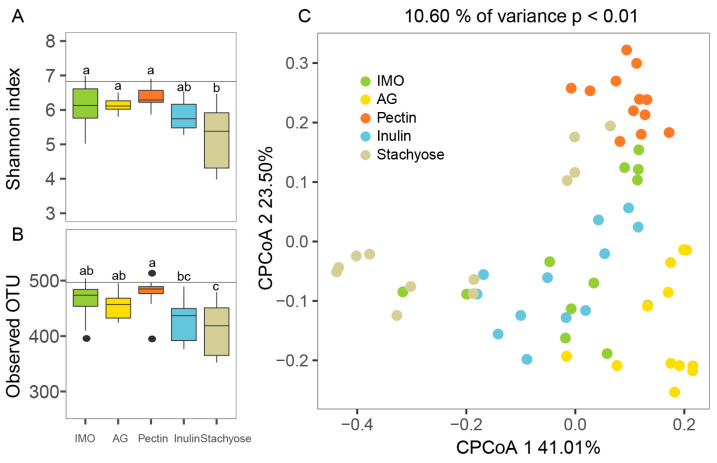
The impact of prebiotics on gut microbiota community diversity of 24 h in vitro culture. (**A**) Shannon index, (**B**) Observed OTU, and (**C**) Constrained PCoA of Bray–Curtis distances. Horizontal lines in (**A**,**B**) indicate the values at 0 h (baseline). Different superscript letters (a–c) indicate significant differences (*p* < 0.05) according to one-way ANOVA with Tukey HSD (*p* < 0.05).

Changes in microbial α-diversity during in vitro culture were assessed using the Shannon index and the number of observed OTUs, which are key indicators of community richness and diversity, respectively. The Shannon index reflects both species richness and evenness within microbial communities, whereas observed OTUs measure the number of detected taxa and primarily reflect species richness [31]. Overall, during the 24 h culture period, all five prebiotic treatments showed a decrease in α-diversity compared with the baseline (0 h), with varying decreases across substrates and being most pronounced in the stachyose group (Figure 1A,B). There were no significant changes in Shannon index in the IMO, AG, pectin, or inulin groups (Figure 1A, *p* > 0.05). In contrast, the Shannon index in the stachyose group was significantly lower than those in the other four groups (Figure 1A, *p* < 0.05). Analysis of observed OTUs further revealed that the IMO, AG, and pectin groups exhibited significantly higher observed OTUs than the inulin and stachyose groups (Figure 1B, *p* < 0.05). Among the five prebiotics treatments, the pectin group displayed the highest Shannon index and observed OTU counts, indicating that pectin supplementation was most effective in maintaining microbial diversity during in vitro culture.

β-diversity analysis was performed using constrained principal coordinate analysis (CPCoA) based on Bray–Curtis distances to evaluate differences in microbial community structure during in vitro culture (Figure 1C). The first and second CPCoA axes explained 41.01% and 23.50% of the total variation, respectively. Distinct clustering patterns were observed among the five prebiotic treatments over the 24 h culture period. Microbial communities from different treatments were clearly separated in the ordination space, indicating that between-group differences were greater than within-group variation. PERMANOVA confirmed that the five prebiotics induced significantly different community structures during fermentation (*p* < 0.01). Along the CPCoA1 axis, notable differences were observed among IMO, stachyose, inulin, and AG groups, whereas along the CPCoA2 axis, the pectin group was clearly separated from the other four treatments.

These results demonstrated that different prebiotic substrates modulated microbial diversity in distinct ways during in vitro culture, resulting in substrate-specific trajectories of community diversity over time.

### 3.2. Microbiota Compositional Changes During In Vitro Culture with Five Prebiotics

**Figure 2:**
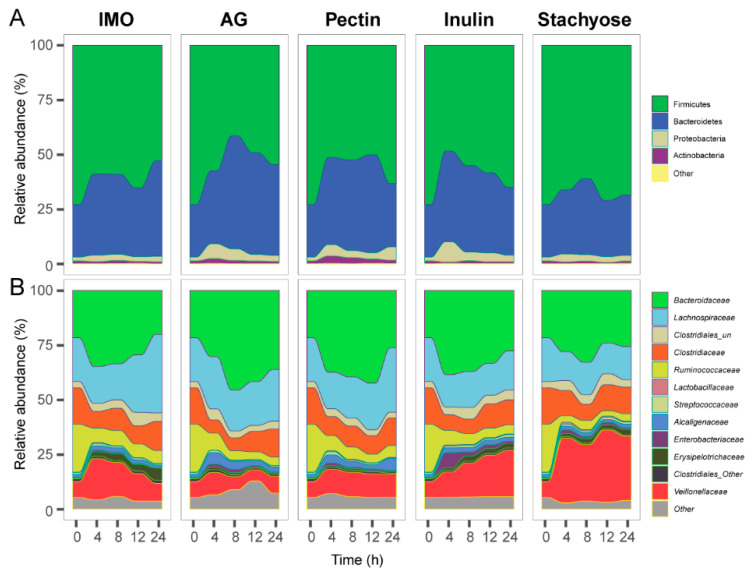
The effects of prebiotics on microbial community composition of 24 h in vitro culture. Microbiota composition is shown at the (**A**) phylum level and (**B**) family level.

To further investigate the effects of the five prebiotics on microbial community composition during 24 h in vitro culture, changes were first analyzed at the phylum level. The microbial communities in all treatment groups were dominated by Firmicutes, Bacteroidetes, Proteobacteria, and Actinobacteria. During the 24 h culture, the relative abundance of Firmicutes in all five groups showed an initial decrease followed by a slight increase, but remained lower at 24 h compared with the baseline (0 h) (Figure 2A). In contrast, Bacteroidetes exhibited an opposite trend, increasing during the early fermentation stage and remaining higher than baseline at 24 h in all groups (Figure 2A). The relative abundance of Proteobacteria displayed different patterns among the treatments. In the AG, pectin, and inulin groups, Proteobacteria abundance initially increased and then decreased over time. In contrast, in the IMO and stachyose groups, no significant changes were observed in Proteobacteria abundance throughout the 24 h culture (Figure 2A, *p* > 0.05).

At the family level, the microbial communities across all five prebiotic treatments were primarily composed of *Bacteroidaceae*, *Lachnospiraceae*, *Clostridiaceae*, *Ruminococcaceae*, and *Veillonellaceae* (Figure 2B). During the 24 h in vitro culture, the relative abundance of *Bacteroidaceae* showed an initial increase followed by a decrease in all groups (Figure 2B). Notably, in the AG group, the abundance of *Bacteroidaceae* at 24 h was significantly higher than at 0 h (Figure 2B, *p* < 0.05). The *Lachnospiraceae* abundance in the IMO group was also significantly higher at 24 h compared with the baseline (Figure 2B, *p* < 0.05). Additionally, *Veillonellaceae* abundance increased significantly in the inulin and stachyose groups after 24 h of culture (Figure 2B, *p* < 0.05).

**Figure 3:**
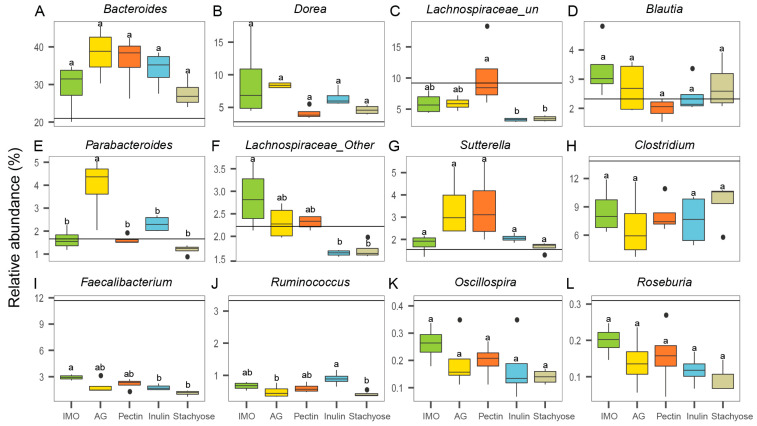
The effects of prebiotics on microbial community composition of 24 h in vitro culture at the genus level. The relative abundance of (**A**) *Bacteroides*, (**B**) *Dorea*, (**C**) *Lachnospiraceae*_un, (**D**) *Blautia*, (**E**) *Parabacteroides*, (**F**) *Lachnospiraceae*_other, (**G**) *Sutterella*, (**H**) *Clostridium*, (**I**) *Faecalibacterium*, (**J**) *Ruminococcus*, (**K**) *Oscillospira*, and (**L**) *Roseburia*. Horizontal lines indicate the relative abundance at 0 h (baseline). Different superscript letters (a,b) indicate significant differences (*p* < 0.05) according to one-way ANOVA with Tukey HSD (*p* < 0.05).

At the genus level, twelve genera with relative abundances greater than 0.1% were presented (Figure 3A–L). The results showed that the relative abundances of *Bacteroides*, *Dorea*, and *Sutterella* increased after 24 h of culture compared with baseline (0 h) in all treatment groups (Figure 3A,B,G). In contrast, the abundances of *Clostridium*, *Faecalibacterium*, *Ruminococcus*, *Oscillospira*, and *Roseburia* decreased after 24 h relative to 0 h (Figure 3I–L). Significant differences among treatments were also observed at 24 h. The relative abundance of *Lachnospiraceae*_un in the pectin group was significantly higher than in the inulin and stachyose groups (Figure 3C, *p* < 0.05). *Lachnospiraceae*_other and *Faecalibacterium* were significantly more abundant in the IMO group than in the inulin and stachyose groups (Figure 3F,I, *p* < 0.05). The AG group exhibited a significantly higher relative abundance of *Parabacteroides* than the other four groups (Figure 3E, *p* < 0.05). Conversely, *Ruminococcus* abundance in the inulin group was significantly higher than in the AG and stachyose groups (Figure 3J, *p* < 0.05).

In summary, different prebiotic substrates induced distinct shifts in microbial composition at the phylum, family, and genus levels during in vitro culture, reflecting substrate-specific modulation of the gut microbiota.

### 3.3. Functional Prediction of Microbial Communities

**Figure 4:**
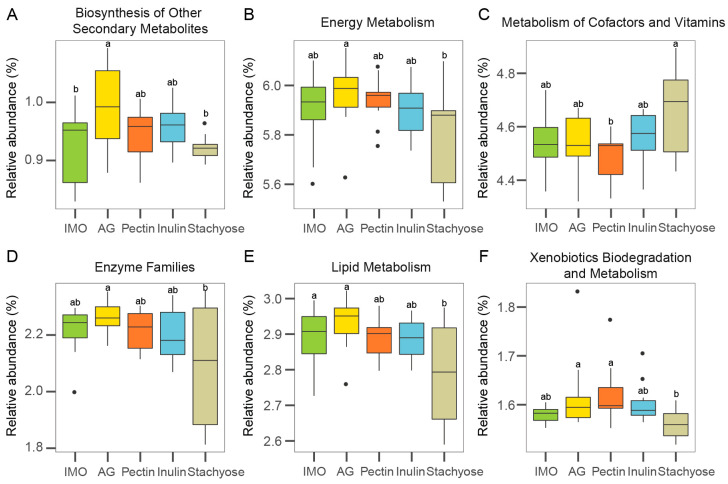
The effects of prebiotics on gut microbiota metabolic functions of 24 h in vitro culture using PICRUSt. The relative abundance of (**A**) Biosynthesis of Other Secondary Metabolites, (**B**) Energy Metabolism, (**C**) Metabolism of Cofactors and Vitamins, (**D**) Enzyme Families, (**E**) Lipid Metabolism, and (**F**) Xenobiotics Biodegradation and Metabolism. Different superscript letters (a,b) indicate significant differences (*p* < 0.05) according to one-way ANOVA with Tukey HSD (*p* < 0.05).

PICRUSt was used to predict microbial functional genes and metabolic pathways from 16S rRNA gene sequences, thereby linking community composition with functional potential. The predicted functional profiles of gut microbiota during in vitro culture are shown in Figure 4A–F. The AG group exhibited a significantly higher abundance of genes related to biosynthesis of other secondary metabolites compared with the IMO and stachyose groups (Figure 4A, *p* < 0.05). Genes involved in energy metabolism, enzyme families, lipid metabolism, and xenobiotics biodegradation and metabolism were also more abundant in the AG group than in the other four treatments, with particularly significant differences compared with the stachyose group (Figure 4B,D–F, *p* < 0.05). In addition, the stachyose group showed a significantly higher abundance of genes associated with metabolism of cofactors and vitamins than the pectin group (Figure 4C, *p* < 0.05).

Overall, these PICRUSt predictions indicate that the five prebiotic substrates exerted distinct influences on the metabolic functional gene profiles of gut microbial communities, with AG supplementation showing the strongest enhancement of metabolic potential during in vitro culture.

### 3.4. Effects of Five Prebiotics on SCFA Production

**Figure 5:**
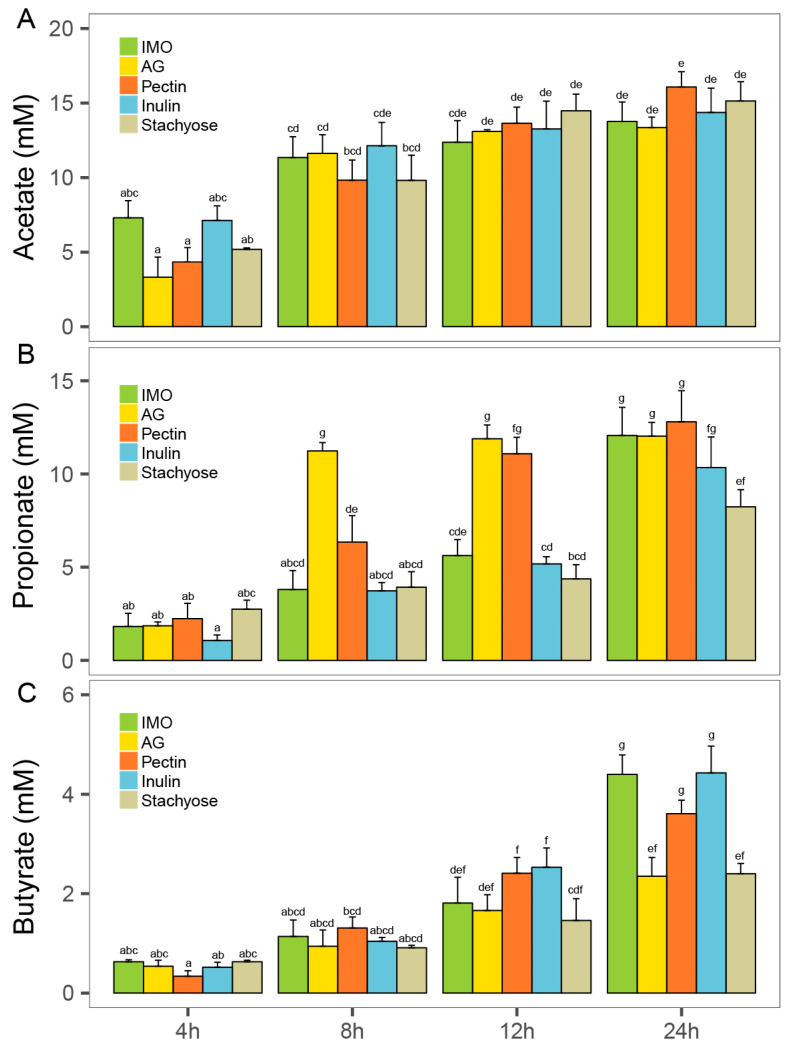
The effects of prebiotics on gut microbiota SCFA production of 24 h in vitro culture. (**A**) Acetate concentration, (**B**) propionate acid concentration, and (**C**) butyrate acid concentration. Different superscript letters (a–g) indicate significant differences (*p* < 0.05) according to one-way ANOVA with Tukey HSD (*p* < 0.05).

Short-chain fatty acids (SCFAs) are major microbial fermentation products in the human gut and play essential roles in host physiology. The concentrations of three major SCFAs (acetate, propionate, and butyrate), were quantified during in vitro culture using liquid chromatography. Overall, acetate and propionate concentrations were higher than butyrate, and all three SCFAs showed a gradual increase over the 24 h incubation in all five prebiotic treatments (Figure 5A–C).

Acetate levels did not differ significantly among treatments during fermentation. At 24 h, the pectin group reached the highest acetate concentration (14.48 ± 1.11 mM) (Figure 5A). For propionate, the AG group exhibited a peak concentration of 11.23 ± 0.45 mM at 8 h, which was significantly higher than the other four groups (Figure 5B, *p* < 0.05). By 24 h, the pectin group had the highest propionate concentration (12.80 ± 0.45 mM), and the IMO, AG, and pectin groups all showed significantly higher propionate levels than the stachyose group (Figure 5B, *p* < 0.05). No significant differences in butyrate concentration were observed among treatments at 8 h (Figure 5C, *p* > 0.05). At 12 h, the pectin and inulin groups showed significantly higher butyrate levels than the stachyose group (Figure 5C, *p* < 0.05). At 24 h, the inulin group exhibited the highest butyrate concentration (4.43 ± 0.38 mM), which was not significantly different from the IMO and pectin groups (*p* > 0.05), but significantly higher than the AG and stachyose groups (Figure 5C, *p* < 0.05).

Correlation analysis revealed substrate-dependent associations between *Lachnospiraceae* abundance and butyrate production (Figure S1). Significant positive correlations were observed in the IMO (r = 0.63, *p* = 0.027) and pectin (r = 0.80, *p* = 0.002) groups, whereas no significant relationships were detected in the AG, inulin, or stachyose treatments (*p* > 0.05).

In summary, different prebiotics stimulated SCFA production to varying extents, with IMO, pectin, and inulin showing the strongest promotion of butyrate formation during in vitro culture.

## 4. Discussion

This study utilized the in vitro culture method to systematically investigated the effects of five prebiotics—isomaltooligosaccharides (IMO), arabinogalactans (AG), pectin, inulin, and stachyose—on the human gut microbiota. The analysis focused on microbial diversity, community composition, functional gene metabolic pathways, and short-chain fatty acids (SCFAs) production. The findings revealed distinct substrate-specific responses, providing new insights into how different prebiotics shape gut microbial ecology and metabolic activity.

The five prebiotics used in this study differ markedly in their chemical structures and degrees of polymerization, which strongly influence how they are utilized by gut microbes. Pectin is a high-molecular-weight polysaccharide characterized by a complex and heterogeneous structure, including a large molecular size, a high degree of branching, and variable levels of methyl esterification [32]. This structural complexity requires specialized enzymatic systems for degradation, resulting in a slower fermentation process compared with simpler structures [33]. In contrast, stachyose is a short-chain raffinose-family oligosaccharide with a well-defined tetrasaccharide structure, which can be selectively utilized by specific gut bacteria, particularly *Bifidobacteria* and *Lactobacillus* [34].Correspondingly, changes in α-diversity reflected these structural differences: pectin treatment most effectively preserved both Shannon index and species richness, whereas stachyose supplementation resulted in the greatest reduction in diversity. These observations are in line with previous in vivo studies. Nie et al. [35] reported that oral administration of dietary fibers to healthy rats for four weeks increased colonic microbial α-diversity, with the apple pectin-treated group showing a trend toward higher Chao-1 index values.

Distinct shifts were observed across taxonomic levels, with more substrate-specific effects evident at the family and genus levels. *Lachnospiraceae*, a dominant family within the Firmicutes that includes numerous butyrate-producing taxa [36], showed increased relative abundance following IMO and pectin supplementation. Members of *Lachnospiraceae* play crucial roles in maintaining colonic health by producing short-chain fatty acids, particularly butyrate, which supports epithelial barrier integrity and exerts anti-inflammatory effects [37]. Arabinogalactans (AG) supplementation increased the relative abundance of *Bacteroidaceae* and *Bacteroides*, both of which are central players in complex carbohydrate degradation and the production of acetate and propionate [38]. *Bacteroides* species possess extensive repertoires of carbohydrate-active enzymes, enabling them to efficiently utilize structurally diverse polysaccharides and influence broader microbial networks through metabolic cross-feeding [39]. Inulin and stachyose stimulated *Veillonellaceae*, a family associated with the utilization of lactate and succinate. While *Veillonellaceae* are normal commensals, several members have been linked to pro-inflammatory states, including increased abundance in inflammatory bowel disease [40]. Notably, *Faecalibacterium*, a key anti-inflammatory commensal and major butyrate producer [41], decreased across all treatments but remained highest in the IMO group. *Faecalibacterium prausnitzii* is well known for its role in producing butyrate and secreting anti-inflammatory metabolites that modulate host immune responses [42]. The relatively higher retention of *Faecalibacterium* under IMO treatment suggests that IMO provides a more favorable niche for this beneficial genus.

SCFAs analysis demonstrated that acetate and propionate concentrations were consistently higher than butyrate, with all three acids increasing during culture. Pectin and AG treatments promoted propionate production, whereas IMO, pectin, and inulin significantly enhanced butyrate levels at later fermentation stages. The observed butyrate promotion likely reflects the enrichment of butyrate-producing taxa such as *Lachnospiraceae* [41]. Given butyrate’s roles in supporting colonic barrier integrity, anti-inflammatory signaling, and regulatory T cell differentiation [12], these metabolic outcomes underscore the functional consequences of substrate-driven community shifts.

Taken together, this study demonstrated that different prebiotics exert distinct and substrate-specific effects on gut microbiota in vitro culture. These findings deepen our understanding of microbiota–prebiotic interactions and provide a mechanistic basis for rational selection and combination of prebiotics to modulate gut microbiota toward beneficial configurations. Future work integrating metagenomics, transcriptomics, and metabolomics will enable more precise functional characterization, while extending these observations to animal models and clinical settings will be essential to validate their physiological relevance and therapeutic potential.

## 5. Conclusions

In this study, we systematically evaluated the effects of five prebiotics on human gut microbiota during 24 h in vitro culture. The results demonstrated distinct, substrate-specific impacts on microbial diversity, community composition, functional gene profiles, and SCFA production. Among the prebiotics, pectin most effectively preserved microbial diversity and promoted propionate and butyrate production, while IMO enhanced the relative abundance of *Lachnospiraceae*, and AG increased both *Bacteroidaceae* abundance and microbial metabolic gene levels. SCFAs results further revealed that IMO, pectin, and inulin strongly stimulated butyrate production. Collectively, these findings deepen our understanding of substrate-specific modulation of gut microbiota and offer valuable implications for future microbiome research and prebiotic development.

## Data Availability

The data presented in this study are available on request from the corresponding author due to privacy restrictions.

## Supplementary Materials

The following supporting information can be downloaded at: https://www.mdpi.com/article/10.3390/foods14213774/s1, Figure S1: Pearson correlation analysis between the relative abundance of *Lachnospiraceae* and butyrate under different prebiotic treatments. (A) Isomaltooligosaccharides (IMO), (B) arabinogalactans (AG), (C) pectin, (D) inulin, and (E) stachyose. Shaded areas represent 95% confidence intervals for the regression line. r and p values were calculated using Pearson’s correlation test..

## Abbreviations

The following abbreviations are used in this manuscript:

SCFAs	Short-chain fatty acids
IMO	Isomaltooligosaccharides
AG	Arabinogalactans
