# Supplementary material for: Prebiotic Structural Diversity Shapes Gut Microbial Diversity, Community Composition, and Metabolic Activity In Vitro
Source: Foods. 2025 Nov 4;14(21):3774. doi: 10.3390/foods14213774 (PMC12608862; doi:10.3390/foods14213774)
Supplement: Supplementary file 1 [file foods-14-03774-s001.zip › foods-3948059-supplementary.pdf]

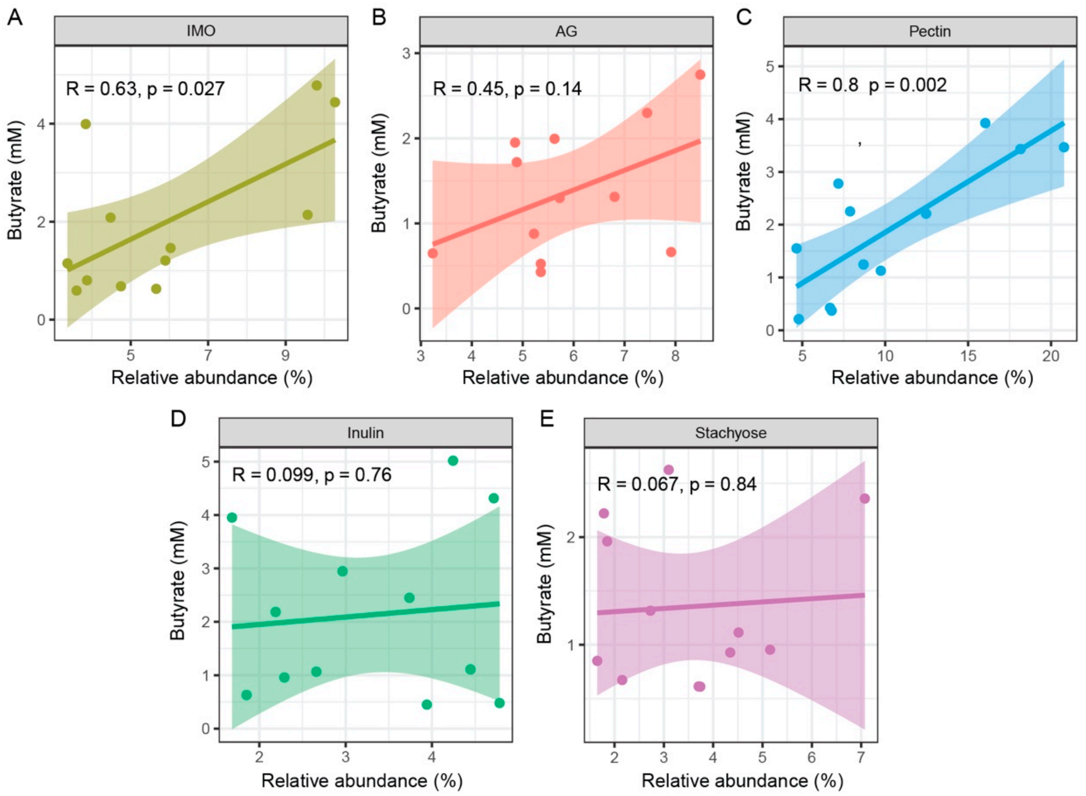

Figure S1. Pearson correlation analysis between the relative abundance of *Lachnospiraceae* and butyrate under different prebiotic treatments. (**A**) Isomaltooligosaccharides (IMO), (**B**) arabinogalactans (AG), (**C**) pectin, (**D**) inulin, and (**E**) stachyose. Shaded areas represent 95% confidence intervals for the regression line. r and p values were calculated using Pearson's correlation test.
